# Lithium intoxication presenting as altered consciousness and arrhythmia with cardiogenic shock: A case report

**DOI:** 10.1097/MD.0000000000013129

**Published:** 2018-11-09

**Authors:** Shun-Ching Chien, Kuan-Ting Liu, Yen-Hung Wu

**Affiliations:** a Department of Emergency Medicine, Kaohsiung Medical University Hospital; b School of Medicine, College of Medicine, Kaohsiung Medical University, Kaohsiung, Taiwan.

**Keywords:** altered mental status, arrhythmia, cardiogenic shock, lithium intoxication

## Abstract

**Rationale:**

Lithium has been used to treat bipolar disorder. Lithium has a narrow therapeutic index, with a therapeutic level between 0.6 and 1.5 mEq/L. The possible complications of lithium overdose include altered mental status, hand tremor, muscle weakness, nausea, vomiting, diarrhea, seizure, syncope, and arrhythmia. Lithium intoxication can be fatal and is difficult to diagnose in patients without a history of lithium intake. The occurrence of serious cardiac arrhythmias is rare in lithium intoxication.

**Patient concerns:**

An 81-year-old man was brought to the emergency department because of consciousness disturbance for 2 days. According to his daughter, he had a history of hypertension and diabetes. Recently, his family also observed slurring of speech and easy choking. The physical examination findings were unremarkable.

**Diagnosis:**

Blood examination only revealed impaired renal function. Twelve-lead electrocardiography revealed sinus rhythm with first-degree atrioventricular block. Chest radiography revealed mediastinal widening. The blood pressures obtained from the 4 limbs showed no significant differences. Subsequently, brain computed tomography revealed no obvious intracranial lesion. A neurologist was consulted, and a recent ischemic stroke could not be ruled out. While in the observation area, his systolic blood pressure decreased to <90 mm Hg and he showed bradycardia, and 12-lead electrocardiography revealed an AV block and long pulse. Contrast-enhanced chest computed tomography revealed no evidence of aortic dissection. Another family member reported a history of lithium intake for bipolar disorder for >30 years. Blood examination revealed a lithium concentration of 2.65 mEq/L.

**Interventions:**

A nephrologist was consulted, and emergency hemodialysis was indicated. Dopamine was administered for his shock status via a right neck central venous catheter.

**Outcomes:**

His lithium level gradually declined after the hemodialysis, and blood pressure and consciousness level improved subsequently. The patient was discharged 9 days later in a stable condition.

**Lessons:**

If an emergency physician encounters a patient with altered consciousness and arrhythmia with cardiogenic shock, the patient's drug intake history should be carefully reviewed to rule out cardiovascular problems on the basis of the patient's clinical condition.

## 1 Introduction

Lithium has been used to treat bipolar disorder. Lithium has a narrow therapeutic index, with a therapeutic level between 0.6 and 1.5 mEq/L.^[1]^ The possible complications of lithium overdose include altered mental status, hand tremor, muscle weakness, nausea, vomiting, diarrhea, seizure, syncope, and arrhythmia. Lithium intoxication can be fatal and is difficult to diagnose in a patient without a history of lithium intake. Here, we report a case of lithium intoxication presenting with altered consciousness and arrhythmia with cardiogenic shock. According to the regulations of the institutional review board of the Kaohsiung Medical University Hospital, ethical approval for this case report article is not required. Informed consent was obtained from the patient for the publication of this case report.

## 2 Case presentation

**Figure 1:**
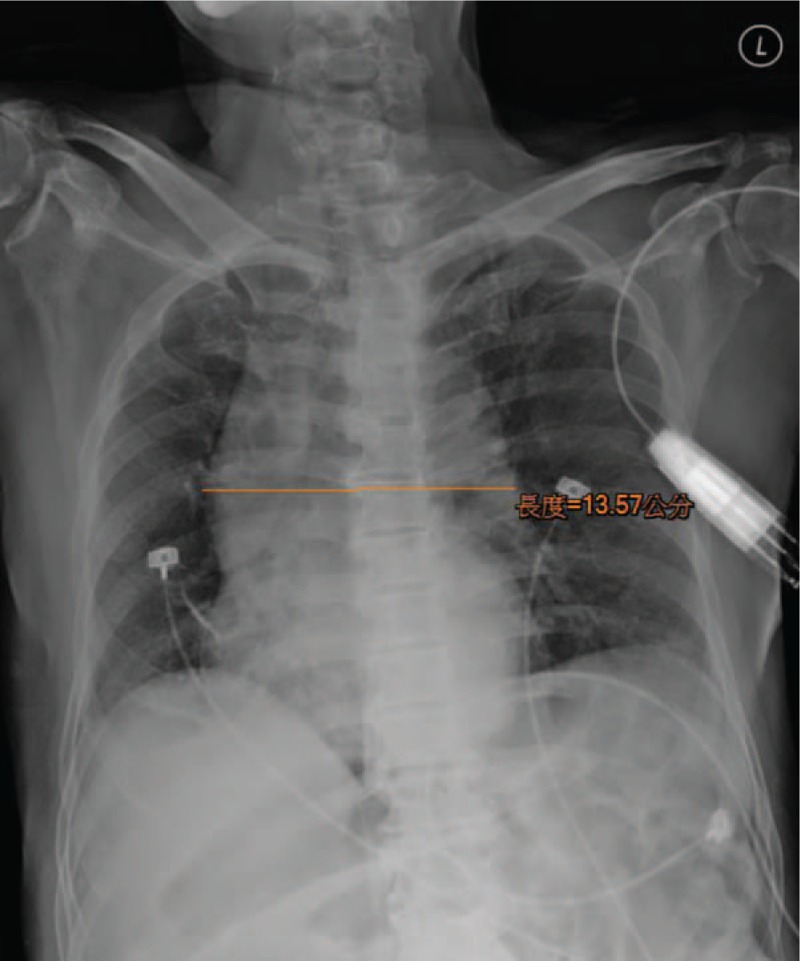
Chest radiograph showing mediastinal widening.

**Figure 2:**
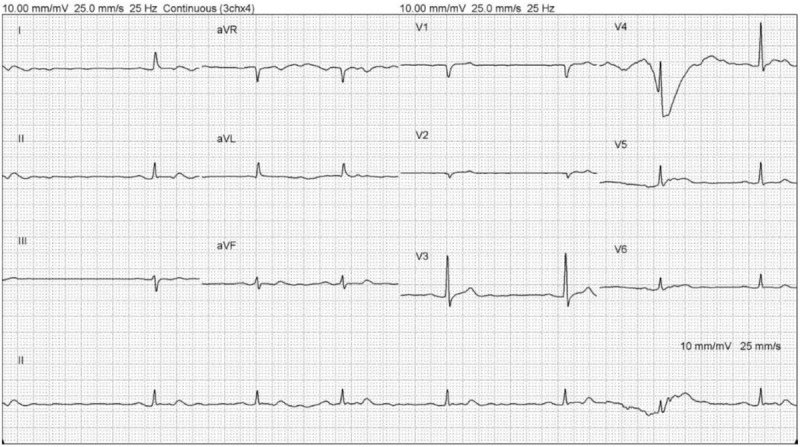
A 12-lead electrocardiogram showing atrioventricular block with bradycardia and long pulse.

An 81-year-old man was brought to the emergency department because of conscious disturbance for 2 days. Upon arrival, his vital signs were as follows: body temperature, 37.9°C; blood pressure, 83/45 mm Hg; heart rate, 71 bpm, and Glasgow coma scale score, 9 (E3V3M3). According to his daughter, he had a history of hypertension and diabetes, with good compliance to medications. He had no history of recent trauma. Recently, his family also observed slurring of speech and easy choking. These physical examination findings were unremarkable. Blood examination, including complete blood count, renal and liver functions, electrolyte, and cardiac enzyme, revealed no elevation of leukocytosis or C-reactive protein level, normal liver function and cardiac enzyme, impaired renal function (creatinine, 2.71 mg/dL), no obvious electrolyte abnormality, and no acidosis. Twelve-lead electrocardiography revealed sinus rhythm with a first-degree atrioventricular (AV) block. Chest radiography revealed mediastinal widening (Fig. 1). Blood pressures obtained from 4 limbs showed no significant differences. Subsequently, brain computed tomography revealed no obvious intracranial lesion. A neurologist was consulted, and a recent ischemic stroke could not be ruled out; thus, admission for further examination was suggested. His blood pressure improved after hydration with normal saline. While in the observation area, his systolic blood pressure decreased to <90 mm Hg and he showed bradycardia, and 12-lead electrocardiography revealed an AV block and long pulse (Fig. 2). Atropine was prescribed, and his blood pressure was elevated for a few minutes but subsequently decreased; thus, dopamine was administered for the shock status via a right neck central venous catheter. Contrast-enhanced chest computed tomography revealed no evidence of aortic dissection. Another family member reported a history of lithium intake for bipolar disorder for >30 years. Blood examination revealed a lithium concentration of 2.65 mEq/L (normal treatment range, 0.5–1.2 mEq/L). Subsequently, a nephrologist was consulted, and emergency hemodialysis was indicated; hence, the patient was transferred to the intensive care unit for further care. His lithium level gradually declined after the hemodialysis, and his blood pressure improved subsequently. He was transferred to the ward after 4 days because of stable hemodynamic status. His consciousness level gradually improved in the ward. He was discharged 9 days later in a stable condition. Neither the patient nor his family reported a history of lithium overdose after discharge because the physician used another drug to control the patient's bipolar disorder.

## 3 Discussion

Lithium is a widely used drug for bipolar affective disorder, depression, and schizoaffective disorder. It has a narrow therapeutic level, and its levels should be monitored carefully and dosage must be adjusted if necessary.

Lithium intoxication is of three types, namely acute, acute on chronic, and chronic. Acute poisoning occurs in individuals who are not treated with lithium. Acute-on-chronic poisoning occurs in patients treated with lithium who take an overdose. Chronic toxicity occurs in patients receiving chronic lithium therapy. Chronic poisoning can occur in patients whose lithium dosage has been increased or in individuals whose renal function has decreased, resulting in an increase in serum lithium levels.^[1]^

The clinical manifestations of chronic lithium intoxication include altered mental status, hand tremor, muscle weakness, nausea, vomiting, diarrhea, seizure, syncope, and arrhythmia. Serious cardiac arrhythmias are rarely seen in lithium toxicity.^[2]^ The cardiac manifestations of lithium toxicity include junctional rhythm, atrial fibrillation, ST-T wave changes, supraventricular tachycardia, and sinus node arrest. The cardiac effects of lithium are observed over a wide range of lithium concentrations at both therapeutic and toxic levels. Its manifestations range from benign ST-segment, T-wave changes to severe manifestations such as sinus node dysfunction, atrial flutter, AV blocks, left anterior hemiblock, right bundle-branch block, ventricular tachycardia, ventricular fibrillation, and sudden cardiac arrest.^[3]^

The possible mechanisms of lithium-induced arrhythmia include the following: lithium causes hyperkalemia by displacing the intracellular potassium ion; lithium is not removed effectively as sodium ions enter cardiac cells during depolarization; the spontaneous rate of depolarization of the sinus node and the conduction velocity in the AV and intraventricular conduction systems are reduced; the response to adrenergic stimulation is reduced; and calcium ion influx interferes in the pacing cells of the sinus node.^[4–6]^

Lithium is concentrated within the thyroid and inhibits thyroid synthesis and release. Thus, lithium can cause hypothyroidism and hypothermia. It can also cause thyrotoxicosis and hyperthermia, as well as hyperparathyroidism and hypercalcemia.^[1]^

Factors that increase the risk of lithium toxicity include renal insufficiency, overdose, volume depletion, infections, decreased effective circulating volume (cirrhosis, congestive heart failure, and nephrotic syndrome), medication interaction (e.g., diuretics and angiotensin-converting enzyme inhibitor), gastroenteritis, and anorexia.^[1,7–9]^

The treatment of chronic lithium intoxication is based on the patient's lithium level and clinical condition. The airway should be protected if consciousness is impaired. If the serum lithium level is >4 mEq/L or the patient has an unstable hemodynamic status and severe neurological symptoms with a serum lithium level of >2.5 mEq/L, hemodialysis should be considered.^[10]^

In this case, the initial impression was acute ischemic stroke when the patient was brought to the emergency department; however, his blood pressure was not elevated, and this was noted by the physician. The mean arterial blood pressure is usually elevated in patients with acute stroke. This may be due to chronic hypertension, which is a major risk factor for ischemic stroke. Type A aortic dissection can present as a stroke, including limb weakness or conscious disturbance with hypotension.^[11]^ Therefore, chest computed tomography was performed because the chest radiograph showed mild mediastinum widening and hypotension, without evidence of aortic dissection. The diagnosis of chronic lithium intoxication was established when another family member reported a history of chronic lithium intake for >30 years (lithium dosage: 600 mg/day), and the lithium level was 2.65 mEq/L.

The patient underwent emergency hemodialysis with indication of an unstable hemodynamic status and severe neurological symptoms, with a serum lithium level of >2.5 mEq/L. The thyroid and calcium levels obtained before hemodialysis were within their normal ranges. Serious cardiac arrhythmias rarely occur, and in this case, the patient presented with AV block with cardiogenic shock. His arrhythmia and consciousness level improved after the lithium level decreased.

If an emergency physician encounters a patient with altered consciousness and arrhythmia with cardiogenic shock, the patient's drug intake history should be carefully reviewed to rule out cardiovascular problems on the basis of the patient's clinical condition.

## Author contributions

**Resources:** Yen-Hung Wu.

**Supervision:** Kuan-Ting Liu.

**Writing – original draft:** Shun-Ching Chien, Yen-Hung Wu.

**Writing – review & editing:** Shun-Ching Chien, Kuan-Ting Liu, Yen-Hung Wu.
